# Hydrophilic Anhydride-Containing Oligomers for Two-Component Hydrogels: From Biopolymer Compatibility to Cytocompatible Gelatin Bioinks

**DOI:** 10.3390/gels12050437

**Published:** 2026-05-16

**Authors:** Julia C. Matros, Katharina E. Wiebe-Ben Zakour, Joana Witt, Michael C. Hacker

**Affiliations:** 1 Heinrich Heine University Düsseldorf, Faculty of Mathematics and Natural Sciences, Institute of Pharmaceutics and Biopharmaceutics, 40225 Düsseldorf, Germany; julia.matros@hhu.de; 2 Department of Ophthalmology, Medical Faculty and University Hospital Düsseldorf, Heinrich Heine University Düsseldorf, 40225 Düsseldorf, Germany; katharinaelisabeth.wiebe-benzakour@med.uni-duesseldorf.de (K.E.W.-B.Z.); joana.witt@med.uni-duesseldorf.de (J.W.)

**Keywords:** two-component hydrogels, gelatin-based bioinks, chitosan-based biomaterial ink, maleic anhydride copolymer, amine–anhydride conjugation, anhydride-containing oligomers, cytocompatible hydrogels, 3D bioprinting

## Abstract

Tissue engineering represents a central strategy in regenerative medicine to restore damaged or missing tissue through structural and functional replacement. In this study, a two-component bioink platform was developed based on amine–anhydride conjugation as a mild crosslinking reaction between synthetic anhydride-containing oligomers (oSMoMA-x) and natural biopolymers. The compatibility of the oligomers with different amine-containing biopolymers, including chitosan, gelatin, and hydrolyzed collagen peptides, was systematically evaluated. To improve cytocompatibility and enable controlled network formation, oSMoMA oligomers with varying anhydride contents were synthesized and characterized, allowing targeted tuning of material properties through comonomer composition. The resulting hydrogels were comparatively assessed with respect to their rheological and physicochemical properties. While hydrogel formation was achieved with all investigated biopolymers, gelatin-based systems exhibited the most favorable characteristics for bioink development. Two gelatin/oSMoMA bioink formulations with distinct gelation behavior were obtained by employing different base catalysts, enabling control over crosslinking kinetics and material properties. Cytocompatibility was comprehensively evaluated using viability assays, demonstrating enhanced metabolic activity of cells encapsulated in gelatin/oSMoMA-3.5 hydrogels compared to established reference systems, with sustained compatibility for up to seven days. Extrusion-based 3D bioprinting was performed using a modified printhead with integrated temperature control to maintain physiological conditions. The bioinks were successfully printed with embedded murine 3T3 fibroblasts, and post-printing analyses confirmed cell proliferation within the hydrogel constructs. Overall, the results demonstrate the broad compatibility of amin–anhydride-crosslinked oSMoMA systems with different biopolymers and highlight gelatin/oSMoMA bioinks as promising cytocompatible materials for stable 3D bioprinting applications in tissue engineering.

## 1. Introduction

Hydrogels represent a versatile class of soft materials that combine high water content with tunable mechanical and physicochemical properties, making them highly attractive for biomedical applications such as drug delivery, tissue engineering, and biofabrication [1,2]. Hydrogels derived from natural polymers, including gelatin and chitosan, are widely investigated due to their inherent biocompatibility, biodegradability, and biofunctionality [3]. However, the limited mechanical stability and restricted tunability of many physically or ionically crosslinked systems remain significant challenges, especially for applications requiring controlled gelation, structural integrity, and long-term cell compatibility [4].

To address these limitations, chemically crosslinked hydrogels have gained increasing attention, as they allow precise control over network architecture and material properties [5]. Among the available chemistries, reactions between nucleophilic amino groups and electrophilic anhydrides provide an efficient and mild crosslinking strategy that is compatible with aqueous environments and physiological conditions. This amine–anhydride conjugation has been successfully employed to crosslink natural polymers using synthetic anhydride-containing macromolecules, yielding hydrogels with adjustable stiffness, degradation behavior, and swelling characteristics [6,7,8]. Importantly, the reactivity of anhydrides enables rapid network formation without the need for external triggers such as light or elevated temperatures, which is advantageous for cell-laden systems.

In the context of extrusion-based 3D bioprinting, bioinks must satisfy a demanding set of requirements, including shear-thinning behavior, controllable gelation kinetics, mechanical stability after deposition, and high cytocompatibility [9]. Two-component hydrogel systems, in which crosslinking occurs upon mixing of reactive components, offer particular advantages by decoupling processing and gelation. Nevertheless, achieving a balance between sufficient reactivity, favorable mechanical properties, and low cytotoxicity remains a central challenge, especially when synthetic crosslinkers are employed [4,10].

This study aims to develop and evaluate a two-component hydrogel platform based on amine–anhydride conjugation for application as a cytocompatible bioink. To this end, a series of anhydride-containing co-oligomers (oligo(stearylacrylate-*co*-acryloyl-morpholine-*co*-maleic anhydride); short: oSMoMA-x) with systematically varied comonomer compositions was synthesized, employing maleic anhydride (MA) to ensure reactivity toward amino-functional biopolymers. Stearylacrylate (SA) was introduced as a lipophilic component to improve mechanical properties, while acryloylmorpholine (AMo) was incorporated to increase hydrophilicity and reduce potential cytotoxicity. The resulting oligomers were comprehensively characterized to establish a library with defined and tunable anhydride contents.

Subsequently, the oSMoMA oligomers were applied as reactive crosslinkers for hydrogel formation with different natural biopolymers, namely gelatin, hydrolyzed collagen peptides (HCPs) and chitosan, enabling a comparative assessment of hydrogel formation and properties. Hydrogel compositions were optimized with respect to stability and cytocompatibility by adjusting oligomer content, base concentration, and solvent composition. Based on these results, a gelatin-based hydrogel was selected and further developed into a bioink suitable for extrusion-based 3D bioprinting. The printability of the bioink and the viability of embedded cells within printed constructs were evaluated over time to demonstrate the suitability of the developed hydrogels for 3D bioprinting applications in tissue engineering.

## 2. Results and Discussion

### 2.1. Anhydride-Containing Oligomers for in Situ Gelling Hydrogels by Anhydride–Amine Conversion

**Figure 1:**
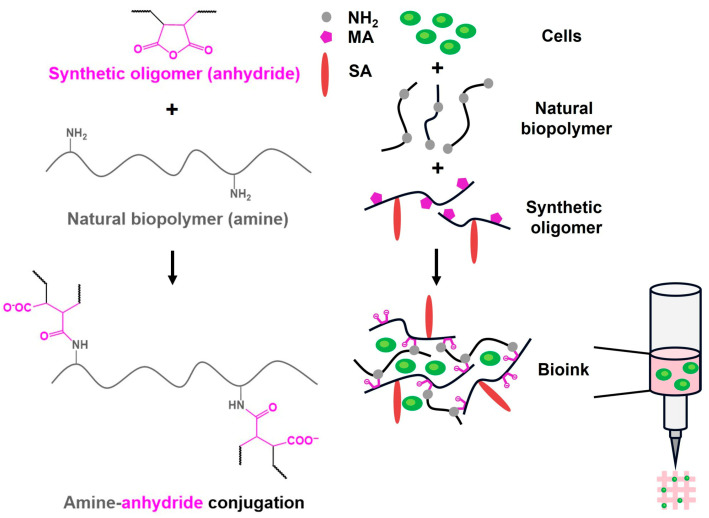
Schematic illustration of hydrogel/bioink formation via amine–anhydride crosslinking. An anhydride-containing synthetic oligomer derived from maleic anhydride (MA) reacts with primary amine groups of a natural biopolymer to form a covalently crosslinked network. Cells are incorporated during mixing to generate a cell-laden bioink that can be extruded to produce hydrogel constructs by 3D bioprinting.

For more than a decade, anhydride-containing oligomers have been systematically investigated by our group as reactive crosslinkers for amine-functional biopolymers, including gelatin, gelatin-derived peptides, and chitosan [6,11,12,13]. These systems exploit the amine–anhydride conjugation reaction to form covalently crosslinked hydrogel networks under mild conditions, enabling precise control over gelation kinetics and mechanical properties (Figure 1). Over successive generations of oligomer design, this strategy has proven to be robust and versatile for the fabrication of hydrogel-based biomaterials.

Earlier oligomer generations, namely oligomers composed of pentaerythritol diacrylate monostearate (PEDAS), *N*-isopropylacrylamide (NIPAM), and maleic anhydride (MA), short oPNMA, demonstrated reliable reactivity toward amino groups and were successfully processed into a variety of two-component hydrogel formats [6,14]. These included prefabricated bulk hydrogels, hollow tubular constructs for nerve regeneration, and crosslinked microparticles for biomedical applications. In these systems, the two reactive components were typically mixed immediately prior to gelation, allowing temporal control over network formation. However, due to the predominantly hydrophobic character of the reactive oPNMA oligomers, their processing required dissolution in water-miscible organic solvents such as dimethylformamide (DMF). The use of DMF was essential to enable homogeneous dissolution of both oligomer and gelatin in mixed solvent systems, thereby ensuring reproducible crosslinking [6]. Nevertheless, the reliance on organic solvents represented a critical limitation for applications involving direct cell contact.

To address this challenge, subsequent studies focused on increasing the hydrophilicity of anhydride-containing oligomers through copolymerization with hydrophilic monomers (h). In particular, the incorporation of polar acrylic comonomers into oP(h)MA-type oligomers significantly improved aqueous compatibility and reduced solvent demand [11,12,15]. This approach enabled the formulation of injectable and cytocompatible hydrogel systems, in which direct embedding of mesenchymal stromal cells during hydrogel formation became feasible. These findings clearly demonstrate that oligomer hydrophilicity is a key determinant for cytocompatibility, without fundamentally compromising anhydride reactivity toward amine-functional biopolymers.

The selection of AMo as the sole hydrophilic comonomer was based on previous systematic evaluations of oP(h)MA systems, in which AMo provided the most favorable balance between enhanced hydrophilicity and controlled copolymerization behavior [11]. In contrast to other hydrophilic monomers, *N*-vinylpyrrolidone and 2-hydroxyethyl acrylate, AMo-containing oligomers maintained a high fraction of intact maleic anhydride functionalities and exhibited predictable molecular characteristics without indications of excessive branching or uncontrolled MA incorporation. At the same time, AMo significantly improved oligomer compatibility with aqueous environments, enabling injectable and cytocompatible hydrogel formulations suitable for direct cell encapsulation. The replacement of PEDAS by SA was motivated by both structural and practical considerations. PEDAS, as a multifunctional diacrylate, can promote branching and locally increased crosslink density within the oligomer backbone, enhancing mechanical reinforcement but also increasing hydrophobicity and solvent demand. In addition, PEDAS has become rarely available from commercial suppliers, limiting its practical suitability for further development and potential translation. In contrast, SA is a monofunctional hydrophobic acrylate that introduces linear hydrophobic segments without additional crosslinking sites, enabling more predictable molecular architecture and tunable mechanical properties. At the same time, its long alkyl chain maintains sufficient hydrophobic contribution for network stability while improving formulation simplicity and compatibility with cytocompatible solvent systems.

**Figure 2:**
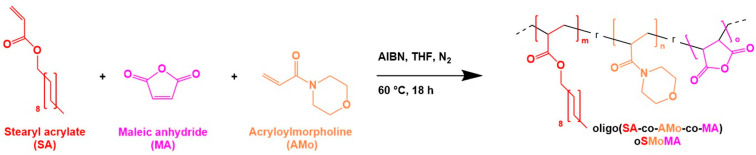
Schematic representation of the free-radical copolymerization of stearyl acrylate (SA), maleic anhydride (MA), and acryloylmorpholine (AMo) to form the oligomeric random copolymer oligo(SA-*ran*-AMo-*ran*-MA) (oSMoMA). The reaction was carried out in dry THF initiated by AIBN under a nitrogen atmosphere at 60 °C for 18 h.

**Figure 3:**
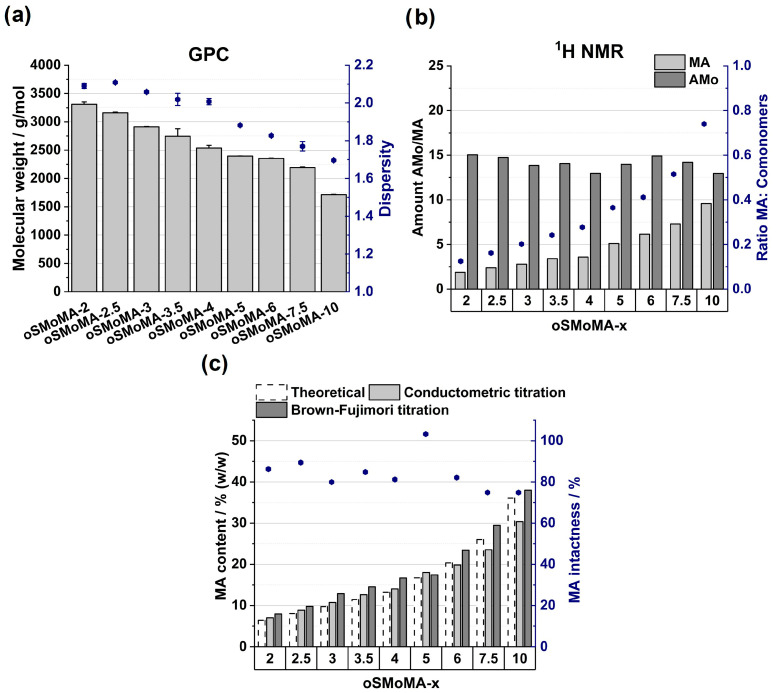
Physicochemical characterization of oSMoMA-x oligomers synthesized with increasing theoretical maleic anhydride (MA) feed ratios (*x* = 2–10). (**a**) Number-average molecular weight (Mn) and dispersity (Đ) determined by gel permeation chromatography (GPC). GPC measurements were performed in triplicate (*n* = 3); bars and points represent mean values and error bars indicate the standard deviation. (**b**) Copolymer composition derived from ^1^H NMR spectroscopy, including calculated MA-to-comonomer ratios. NMR measurements were performed once for each sample. (**c**) Theoretical MA contents in comparison to anhydride contents determined by conductometric titration and Brown–Fujimori titration. Titrations were performed in triplicate (*n* = 3). The obtained mean values were used to calculate MA contents according to the method described by Brown–Fujimori [14]. Since calculated values are reported, no standard deviations are shown.

A series of oSMoMA-x oligomers were synthesized with predefined ratios of MA and AMo in the reaction feed as shown in Figure 2. As desired, the previously established synthesis protocol could be used without changes. Characterization of the oligomers by ^1^H NMR, GPC, and titration methods (conductometric titration and Brown-Fujimori titration) is shown in Figure 3 and revealed that the comonomers were integrated in the copolymers and number average molecular weight was below 3.5 kDa, substantiating the oligomeric character of the copolymers and renal eliminability [16]. Analysis of ^1^H NMR spectra yielded comonomer incorporation ratios and confirmed that increasing the relative anhydride feed resulted in a higher incorporation of anhydride units and an increased MA/AMo ratio. An exemplary NMR spectrum is shown in the Supplementary Material (Figure S1). The theoretical ratio in oSMoMA-10 is 1:1; however, the experimentally determined ratio deviated from this theoretical value, indicating a comparatively higher incorporation of AMo. This behavior was observed for all synthesized compositions and may be attributed to the higher radical polymerization reactivity of AMo as compared to MA, for which the electron-withdrawing carbonyl group and cyclic structure reduced reactivity [17]. Molecular weight decreased with increasing MA feed, consistent with the lower molecular weight contribution of MA relative to AMo. Additionally, dispersity values decreased with higher MA fractions, suggesting a narrower molar mass distribution. Due to the susceptibility of anhydride groups to hydrolysis, the fractions of chemically intact anhydrides were quantified by the combination of two titration methods. This analysis revealed increasing anhydride contents with rising theoretical MA feeds and anhydride integrity exceeded 75% for all oligomers. These values compare favorably with previously reported oligomer systems, which exhibited anhydride integrities in the range of approximately 66–72% [13].

The polymerization process was designed to yield low-molecular-weight oligomers in a defined kDa range suitable for biomedical applications, particularly with respect to renal clearance and overall biocompatibility. As expected for free-radical polymerization, molecular weight can be tuned via monomer concentration and initiator loading. A key factor governing copolymer composition is the relative reactivity of the comonomers. In previous studies, AMo was identified as a suitable hydrophilic comonomer that copolymerizes efficiently with MA; however, preferential incorporation and local sequence enrichment can occur due to differences in monomer reactivity ratios. These effects were mitigated by appropriate adjustment of reaction conditions.

Within the oSMoMA series, a systematic dependence of the number-average molecular weight (M_n_) on the MA feed ratio was observed, with increasing MA content leading to lower molecular weight. This trend is consistent with both the lower molar mass of MA and its influence on copolymerization kinetics, including its tendency to favor alternating incorporation with electron-rich comonomers. The dispersity values remained within a narrow range, indicating a reproducible free-radical polymerization process with limited chain-length heterogeneity. Reproducibility was confirmed by consistent molecular weight distributions and copolymer compositions across the series; repeated syntheses of selected formulations showed batch-to-batch variations in average molecular weight of less than 10%. Residual monomer content is expected to be minimal due to purification by repeated precipitation and drying, which is further supported by consistent ^1^H NMR spectra indicating effective monomer incorporation and removal of non-reacted species.

The fraction of intact anhydride groups below 100% is attributed to the inherent moisture sensitivity of cyclic anhydrides. Partial hydrolysis during work-up, purification, storage, or analytical handling can convert anhydride moieties into the corresponding dicarboxylic acid structures. Thus, the non-intact fraction does not necessarily represent loss of incorporated MA units, but rather ring opening of reactive anhydride groups. To counteract this effect, the oligomers were subjected to heat treatment under dry conditions prior to use, which is expected to promote dehydration and partial re-formation of cyclic anhydride functionalities.

### 2.2. Two-Component Hydrogel Systems Based on oSMoMA and Gelatin, Hydrolyzed Collagen Peptides, or Chitosan

With this series of oSMoMA oligomers at hand, the first hypothesis that these oligomers provide sufficient hydrophilicity to form two-component hydrogels with three different biopolymers can be tested. The three biopolymers are gelatin, hydrolyzed collagen peptides (HCP) that show no sol–gel transition, and chitosan, and require different conditions to form covalent hydrogel networks by anhydride-amine conversion. For each biopolymer, several formulations were initially investigated, and the most suitable variant was selected based on the physicochemical properties of the resulting hydrogels.

**Figure 4:**
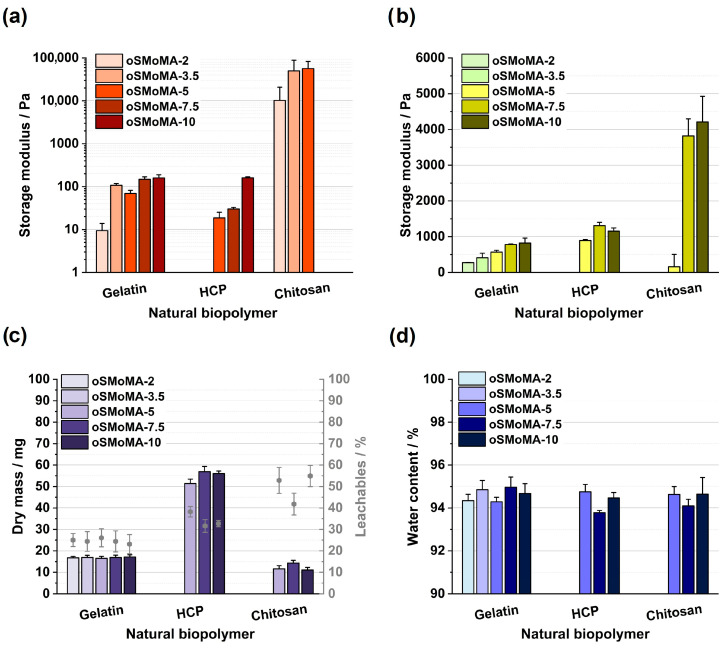
Rheological and physicochemical characterization of prefabricated hydrogels formed from different natural biopolymers (gelatin, HCP, and chitosan) crosslinked with oSMoMA-x oligomers (*x*: 2–10) after washing and equilibrium swelling in PBS. (**a**) Storage modulus (G′) measured after 1000 s in the time sweep (logarithmic scale). (**b**) Storage modulus (G′) of the hydrogel determined under defined oscillatory test conditions (linear scale). (**c**) Dry mass of the resulting hydrogels and corresponding leachables. (**d**) Water content of the hydrogels. All measurements were performed in triplicate (*n* = 3); bars represent mean values and error bars indicate the standard deviation. Missing datasets correspond to formulations that did not yield stable or analyzable hydrogels.

With a selection of oligomers spanning the available range of anhydride contents, namely oSMoMA-2, -3.5, -5, -7.5, and -10, gelation profiles were investigated with biopolymer solutions that had been optimized for concentration and reaction conditions. The gelation behavior of two component mixtures of natural biopolymers with oSMoMA oligomers was evaluated by oscillatory rheology using the storage modulus (G′) as an indicator of network formation and mechanical stability. For comparison, the G′ values at 1000 s were analyzed (Figure 4a).

Across all the systems investigated, an increase in anhydride content of the oligomer led to higher storage moduli. In general, more anhydride groups result in a higher crosslinking density and thus stiffer hydrogels. However, depending on the polymer system, the increase in G′ was not always linear, suggesting that at higher anhydride contents, saturation of available amine groups and mobility limitations may restrict the formation of further crosslinks. For gelatin-based formulations, all oligomers (oSMoMA-2 to oSMoMA-10) resulted in stable hydrogels. At 1000 s, G′ values ranged between 10 and 150 Pa depending on the anhydride content of the oligomer. The highest storage modulus values under homogeneous gelation conditions were obtained for oSMoMA-3.5 and oSMoMA-5, indicating the formation of the most mechanically stable hydrogels within the tested series. At higher theoretical anhydride contents (≥7.5), partial precipitation occurred during preparation, which likely contributed to artificially elevated G′ values due to solid particulates rather than homogeneous network formation. As observed in past studies, above a certain limit of anhydride density per oligomer, solubility limits can be exceeded during the initial phase of the crosslinking reaction and/or inhomogeneously crosslinked gels are formed.

HCP-based hydrogels formed only with oligomers of MA feeds ≥ 5; lower MA contents did not result in gelation within the measured period. At 1000 s, G′ values were comparable to those of gelatin (approximately 20–150 Pa), even though the final biopolymer concentrations differed due to the distinct formulation compositions. Previous experiments have indicated that HCP-based formulations can exhibit slower gelation at early time points compared to gelatin-based mixtures. Similar to gelatin-based formulations, increasing MA content enhanced the final storage modulus of HCP-based gels. The comparable end-point stiffness of gelatin (3.3% (*w*/*v*)) and HCP (18.8% (*w*/*v*)) suggests a similar effective crosslinking density at 1000 s, despite differences in gelation kinetics. Both systems exhibited mechanical strength suitable for soft tissue applications [18]. Chitosan hydrogels exhibited substantially higher storage moduli than gelatin- and HCP-based gels. At 1000 s, G′ values reached approximately 10^4^–10^5^ Pa. Only oligomers with an MA feed ≤ 5 were measurable, as higher functionalization led to moduli exceeding the instrument’s reliable measurement range. The pronounced stiffness of chitosan hydrogels can be attributed to the high density of primary amine groups and their efficient participation in crosslinking reactions [12]. Relatively large standard deviations were observed, suggesting mixing inhomogeneities due to fast viscosity increase.

The three tested biopolymers have vast applications in biomedical applications, including musculosceletal regeneration, and exhibit interesting biomedical properties [19]. Using the strategy of two-component crosslinked hydrogel formation with anhydride-containing oligomers of the oSMoMA type allows one to obtain storage moduli ranging from 10 to 10^5^ Pa providing compatibility with a range of soft and hard tissue applications and illustrating the potential of these systems for biomaterial ink or even bioink development. This tunability is highly relevant for biofabrication and injectable hydrogel systems, where mechanical properties must be adapted to the target tissue environment and processing requirements [20]. In addition, this platform provides the opportunity for facile covalent modification with biologically active heterobifunctional amines as demonstrated in previous studies with previous generations of anhydride-containing oligomers [21,22].

In addition to rheological analysis of the crosslinking reaction, hydrogels were fabricated and subjected to equilibrium swelling in water/PBS and analyzed using a plate–plate geometry (Figure 4b). This analysis of pre-fabricated hydrogels revealed higher storage moduli of peptide-based hydrogels as observed at the end of the monitored crosslinking reaction. For chitosan-based gels, the equilibrium swollen and sol fraction-depleted gel showed lower storage moduli when compared with the crosslinking experiments. Again, as expected, gel moduli increased with increasing anhydride content of the oligomers. This trend is consistent with previous studies by Kascholke et al., who reported a similar increase in G′ for short-chain gelatin combined with oPDMA oligomers, particularly from oPDMA-3.5 to oPDMA-5.5. The highest G′ values were observed for chitosan hydrogels with oSMoMA-7.5 and oSMoMA-10. HCP-based hydrogels showed a maximum in mechanical strength at oSMoMA-7.5.

Across various two-component networks composed of gelatin or partially hydrolyzed collagen and anhydride-containing oligomers, we consistently observed that increasing anhydride content accelerates crosslinking kinetics. However, this relationship is not linear across the full compositional range. Instead, an optimal window can be identified in which increasing anhydride content enhances network formation, as reflected by higher degrees of crosslinking and increased storage moduli (G′) of equilibrium-swollen gels. Beyond this optimum, further increases in anhydride content lead to less homogeneous network structures and reduced stability. This behavior is attributed to rapid initial crosslinking, which limits polymer chain mobility and results in incomplete amine–anhydride conversion and a lower effective crosslink density.

In addition to oligomer composition, the effective crosslink density is strongly influenced by the density and accessibility of primary amine groups within the respective biopolymer. Gelatin provides lysine-derived amine functionalities typically in the range of approximately 0.2–0.4 mmol g^−1^, whereas chitosan exhibits substantially higher amine densities in the mmol g^−1^ range depending on its degree of deacetylation. In contrast, hydrolyzed collagen peptides contain comparable functional groups but reduced chain length, which limits network connectivity despite potentially improved accessibility. These differences are expected to modulate gelation kinetics and the resulting viscoelastic properties at comparable oligomer compositions.

Differences between biopolymers further modulate this behavior, as variations in molecular structure influence the effective crosslink density and resulting viscoelastic properties. Importantly, the oligomer design does not aim to generate classical telechelic crosslinkers with defined end-group functionality, but rather a statistical distribution of reactive anhydride groups along the oligomer backbone.

In addition to anhydride content, parameters such as base concentration and the sequence distribution of anhydride functionalities along the oligomer backbone (often described as molecular anhydride distribution, MAD) have been identified as key factors influencing network formation. Ongoing studies aim to systematically elucidate these effects; until then, controlled experimental series, as presented here, provide an empirical basis to describe the influence of anhydride content and polymer molecular weight on hydrogel properties.

The dry mass and extractable fractions of the hydrogel disks were determined from gravimetric analysis during gel preparation (Figure 4c). Chitosan-based gels exhibited the lowest dry mass and the highest extractable fraction, with nearly 50% of the theoretical solid content removed during processing. HCP-based gels showed the highest dry mass, accompanied by extractable fractions of approximately 30%. Gelatin-based gels displayed the lowest extractable fractions, consistently below 30%. These differences are likely a result of biopolymer-related differences in crosslinking density of the gel networks. Chitosan, gelatin, and gelatinous peptides differ in both the spacing and distribution of amine groups required for the amine-anhydride conjugation crosslinking reaction. Consequently, the compatibility between functional group positioning and the applied oligomers may vary between biopolymers. A lower crosslinking density, as suggested for chitosan-based gels, would lead to increased solubility and thus a greater loss of solid material during washing. For HCP- and chitosan-based hydrogels, oSMoMA-7.5 appeared to yield the most favorable properties. As in previous studies, we consider gels with extractable fractions below 40% sufficiently stable [6]. Based on this criterion, all analyzed gelatin- and HCP-based gels fall within the category of stable hydrogel systems.

No discernible trend was observed in the water content of the hydrogels. Across all formulations, irrespective of the oligomer type or natural biopolymer used, the water content ranged between 93% and 95% (Figure 4d). According to established definitions [23,24], all hydrogels obtained in this study exhibit a high water content.

The comparable dry mass and equilibrium water content across formulations, despite varying MA content, indicate a plateau regime of robust network formation. As biopolymer-to-oligomer ratios were adjusted for each system, direct stoichiometric comparison is limited. Within this regime, increased MA content does not proportionally affect swelling, likely due to constraints in chain mobility and reactive group accessibility, while hydrophilic AMo units promote water uptake. Notably, at high water contents (>85%), small absolute differences mathematically correspond to large changes in fold swelling, masking compositional effects. Accordingly, gravimetric parameters are less sensitive than rheological measurements, which better resolve underlying differences in network structure.

The formulations that were considered best among all investigated mixtures for the biopolymers gelatin, HCP and chitosan are summarized in Table 1.

Overall, these results demonstrate that oSMoMA-x oligomers provide a versatile crosslinking platform that can be adapted to biopolymers with markedly different chemical and physical characteristics. At the same time, the findings highlight that optimal oligomer composition and formulation parameters are strongly biopolymer-specific. Chitosan-based hydrogels formed mechanically stronger networks with slower gelation kinetics, whereas HCP- and gelatin-based gels exhibited lower stiffness, making them more suitable for applications such as 3D bioprinting. Mechanical properties play a critical role in biofabrication, as cell morphology, adhesion, proliferation, and differentiation are strongly influenced by hydrogel stiffness. For each cell type, an optimal mechanical environment must be identified. Hydrogels with insufficient stiffness may lack printability due to unfavorable diffusion behavior, while excessively stiff networks can negatively affect cell viability [25]. Fine-tuning of hydrogel properties can be achieved by adjusting the anhydride content of the oligomers. However, not all natural biopolymers are equally compatible with every oligomer formulation.

### 2.3. Bioink Formulations with Gelatin and oSMoMA

Gelation kinetics play a critical role in extrusion-based 3D printing, as they directly influence processability and printability. In this context, the choice of base system is particularly important. In addition to the recently established organic base triethanolamine (TEOA) [11], the inorganic base dipotassium hydrogen phosphate (K_2_HPO_4_) was considered as it was previously shown to implement a more sustained gelation profile [26]. In preliminary experiments, concentrations of 6% TEOA and 10% K_2_HPO_4_ were established. Gelation profiles and storage moduli of the formulations with different concentrations of TEOA and K_2_HPO_4_ are provided in the Supplementary Material (Figures S2 and S3). Direct comparisons with previously reported printable two component formulations [26] are limited, as these were composed of a more hydrophobic oligomer system (oPNMA) requiring DMF as solvent and were based on HCP (Imagel^®^) instead of gelatin. Nevertheless, oSMoMA/gelatin hydrogels containing K_2_HPO_4_ demonstrated slower gelation kinetics than with TEOA as base. The opportunity to control gelation kinetics by the choice of base provides an interesting feature to adjust extrusion properties and deposition characteristics of a potential bioink for 3D bioprinting. The gelatin-based formulations with oSMoMA in DMSO provide a potentially cytocompatible environment that cannot be achieved when DMF is necessary to dissolve the oligomers and maintain solubility of oligomer and biopolymer upon mixing. Consequently, promising gelatin/oSMoMA formulations were characterized based on rheological properties, dry weight, extractable fraction, water content, crosslinking degree, and cell viability. In the upcoming datasets, selected data for gelatin formulations with TEOA is reproduced from the previous section. However, the formulation space is expanded by including additional oSMoMA-x oligomers and a different base. Furthermore, a more detailed investigation of parameters relevant for bioprinting is presented.

**Figure 5:**
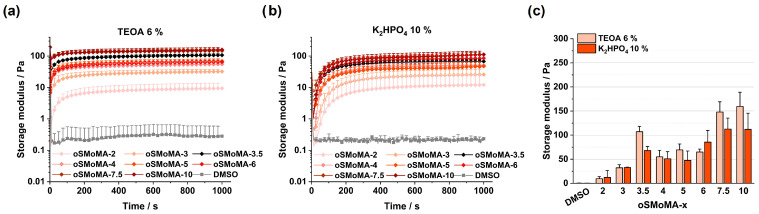
Time-dependent development of the storage modulus (G′) during hydrogel formation for oligo(SA-*co*-AMo-*co*-MA) (oSMoMA-x) formulations with different MA content. (**a**) Crosslinking in the presence of 6% TEOA. (**b**) Crosslinking in the presence of 10% K_2_HPO_4_. In both systems, rapid gelation is observed followed by a plateau in storage modulus, indicating completion of network formation. (**c**) Comparison of the final storage modulus values for the different oSMoMA-x compositions obtained with TEOA (6%) and K_2_HPO_4_ (10%) after 1000 s. DMSO serves as a non-crosslinking control. All measurements were performed in triplicate (*n* = 3). Data points and bars represent mean values, and error bars indicate the standard deviation.

Rheological monitoring of candidate formulations with TEOA (Figure 5a) demonstrates a clear correlation between MA content and hydrogel stiffness upon initial gelation, as reflected by increasing storage modulus (G′) values with higher MA incorporation. This trend is also reflected in the corresponding bar diagram (Figure 5c), which summarizes the storage modulus values after 1000 s and clearly reproduces the differences observed in the time-resolved measurements. This behavior is independent of the base used. Nevertheless, the choice of base has a pronounced influence on both gelation rate and final mechanical properties. Systems formulated with 6% TEOA consistently show faster gelation and higher plateau G′ values than those prepared with 10% K_2_HPO_4_ (Figure 5b). Comparable effects have been reported previously for hydrogel systems prepared with different bases, where the use of K_2_HPO_4_ instead of organic bases, e.g., NMPO, resulted in delayed gelation and lower storage moduli [26]. Since TEOA and NMPO are organic bases, whereas K_2_HPO_4_ is inorganic, the slower reaction kinetics observed for K_2_HPO_4_-containing systems may be attributed to differences in reaction mechanisms and base reactivity. Despite this kinetic disadvantage, K_2_HPO_4_ remains a suitable alternative base. All oligomer-containing formulations exhibit substantially higher storage moduli compared to the DMSO reference without oligomer, confirming their positive contribution to network formation. Interestingly, hydrogels based on oSMoMA-3.5 show unexpectedly high G′ values for both base systems, exceeding those predicted by MA content alone. This indicates the existence of an optimal MA concentration for this biopolymer system, potentially arising from favorable oligomer polarity or optimal spacing of anhydride units. In contrast, results obtained for oSMoMA-7.5 and oSMoMA-10 should be interpreted cautiously due to partial precipitation during sample preparation.

**Figure 6:**
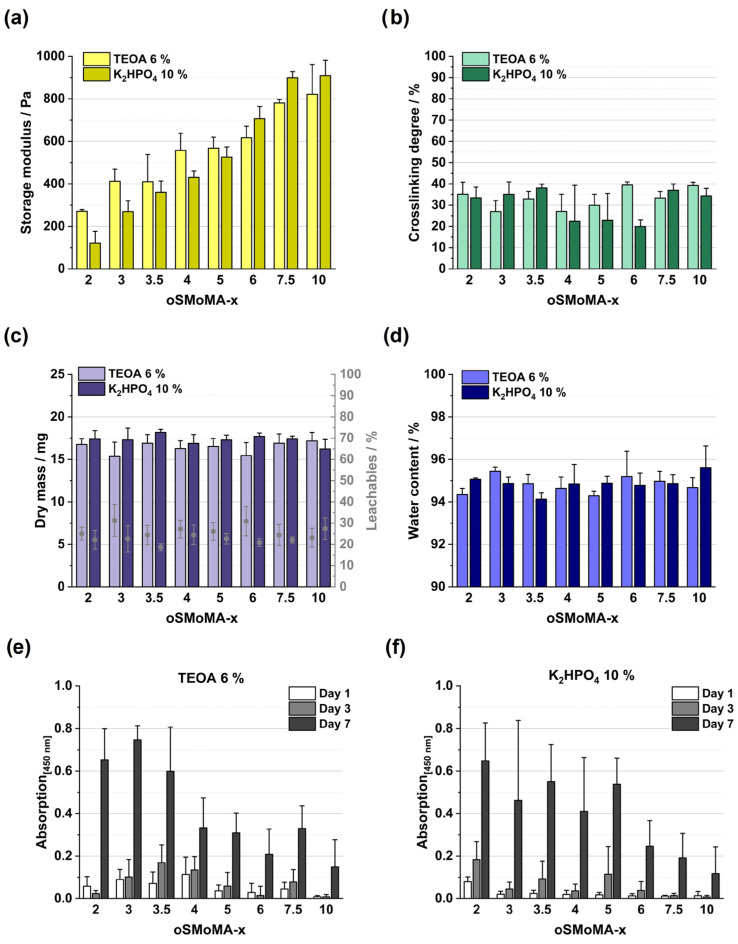
Influence of base component (TEOA 6% vs. K_2_HPO_4_ 10%) and MA feed ratio (oSMoMA-x, *x* = 2–10) on the physicochemical and biological properties of gelatin-based hydrogels: (**a**) Storage modulus (G′); (**b**) Crosslinking degree (%); (**c**) Dry mass and corresponding leachable fraction (%); (**d**) Water content (% (m/m)); (**e**) Metabolic activity of encapsulated cells in hydrogels prepared with TEOA 6%, determined as absorbance at 450 nm on days 1, 3, and 7; (**f**) Metabolic activity of encapsulated cells in hydrogels prepared with K_2_HPO_4_ 10%, determined as absorbance at 450 nm on days 1, 3, and 7. For panels (**a**–**d**), measurements were performed in triplicate (*n* = 3). For cell viability experiments (**e**,**f**), measurements were performed in quadruplicate (*n* = 4). Data is presented as mean values with error bars indicating the standard deviation.

The formulations were processed into pre-fabricated equilibrium-swollen hydrogel discs and analyzed by frequency-dependent oscillatory rheology. The storage moduli of the hydrogels (Figure 6a) follow the same order as identified during time-resolved gelation, indicating that the mechanical differences established during network formation are preserved after post-processing. Quantification of the crosslinking degree by TNBS assay (Figure 6b), which quantifies the fraction of gelatin amino groups that reacted with anhydride functionalities of the oligomers, revealed comparable degrees of crosslinking in the range of approximately 30–40% independent of formulation composition. Compared to literature data reported for oPNMA-based hydrogels with HCP (50–80%), oPNMA with gelatin type B (Bloom 50; 30–75%) [6], and oPDMA with HCP (40–65%), the crosslinking degrees obtained for the oSMoMA/gelatin hydrogels are generally lower. However, a direct comparison is not feasible due to substantial differences in oligomer chemistry, lower molecular weight of the biopolymer component, and solvent systems used for hydrogel preparation. The formulations evaluated here are optimized for cytocompatibility and used DMSO as an organic component in minimized concentrations, while previous systems used DMF as organic solvent and were optimized for amine–anhydride conversion. Among the investigated formulations, oSMoMA-3.5 exhibits particularly promising results for both bases, alongside oSMoMA-7.5 and oSMoMA-10. While a higher degree of crosslinking can be indicative of increased hydrogel stability due to a greater number of amine-anhydride conjugation sites, the conversion obtained here resulted in effective gelatin crosslinking and stable hydrogel network. Finally, no consistent trend can be identified with respect to the choice of base, as certain oligomers exhibit higher crosslinking degrees when prepared with 6% TEOA, whereas others perform better with 10% K_2_HPO_4_. Therefore, these results do not allow a definitive conclusion regarding the superior suitability of either base.

Gravimetric analysis of gel disc dry mass, extractable fractions, and equilibrium water content (Figure 6c) revealed no pronounced differences among the investigated hydrogel formulations. Across all compositions and oligomer types, extractable fractions remained below 40%, indicating efficient network formation and a high degree of structural integrity of the hydrogel plates. The absence of substantial variation in extractables suggests that amine–anhydride conjugation proceeds reliably over the examined range of component concentrations and oligomer compositions, resulting in consistent incorporation of the reactive oligomers into the hydrogel network. Similarly, comparable dry mass and water content (Figure 6d) values demonstrate that network density and swelling behavior are maintained within a narrow and controlled window. Collectively, these findings confirm that the two-component formulation strategy is robust and that all investigated oligomer variants are effectively integrated into stable hydrogel matrices. Based on gravimetric parameters alone, no single formulation exhibits a distinct advantage, underscoring the versatility and reproducibility of the developed platform.

A critical prerequisite in the development of bioinks for cell-laden applications is the comprehensive evaluation of biocompatibility [27]. In contrast to prefabricated scaffolds and biomaterial inks, bioinks are processed in direct contact with living cells during mixing, extrusion, and network formation, exposing cells to reactive groups, solvents, and base components. Even minor formulation changes may therefore significantly affect cell survival, metabolic activity, and long-term proliferation. Consequently, beyond physicochemical characterization, systematic biological assessment is indispensable to evaluate the suitability of two-component hydrogel systems for biofabrication [11]. Biocompatibility information of the oligomer solvent DMSO and oligomer solutions in DMSO is provided in the Supplementary Materials (Figure S4). To quantify cytocompatibility and monitor cellular response within the hydrogel matrix, metabolic activity assays based on water-soluble tetrazolium salts (WST) were employed. The WST-8 assay is widely used for three-dimensional cell culture systems, as the water-soluble formazan product formed upon mitochondrial reduction remains in the supernatant and can be quantified colorimetrically without additional solubilization steps. This approach enables indirect determination of viable cell count and proliferation within hydrogel constructs over time. By applying this standardized methodology, the influence of oligomer type, component concentrations, and formulation parameters on cell viability could be systematically assessed under conditions relevant for bioink processing and 3D bioprinting.

As illustrated in Figure 6e,f, cell number increases up to day 7 for all formulations, regardless of the oligomer or base used. Quantitative interpretation of WST data in cell-laden hydrogels is inherently more challenging than conventional 2D calibration in well plates, as diffusion limitations, matrix absorption of reagents or formazan product, and light scattering effects can influence signal intensity. Consequently, calibration curves generated under 2D conditions cannot be directly transferred to cell-laden 3D samples; therefore, the presented graphs illustrate the measured absorbance per well rather than calculated absolute cell numbers and are interpreted comparatively. The most pronounced increase in enzymatically reduced dye occurs between day 3 and day 7 for all hydrogels, indicating successful 3D cell culture with proliferation of the encapsulated cells. This suggests that the cells were effectively embedded within the hydrogel matrix and were able to receive biochemical and physical signals from both the matrix and neighboring cells. Initially, cells were suspended in a precursor gelatin solution and subsequently immobilized through a crosslinking reaction, forming the hydrogel network. Hydrogels mimic the extracellular matrix (ECM), providing both structural support and signaling cues necessary for cellular function. For gelatin-based hydrogels, specific sequences such as RGD promote cell adhesion and spreading. The mechanical properties of the hydrogel, including stiffness and network structure, influence cell migration and distribution prior to proliferation. Ideally, hydrogel degradation should match the rate of cell proliferation to maintain structural stability [28]. Figure 6e,f demonstrate a composition-dependent effect of oligomer type on cell compatibility. For both bases investigated, metabolic activity decreased for oligomers from oSMoMA-4 onward compared to oSMoMA-2, -3, and -3.5. The highest absorbance values were consistently observed for oSMoMA-2, -3, and -3.5, and this trend was already apparent on day 1, suggesting an early influence of oligomer composition on cellular response. The reduced metabolic activity at higher anhydride contents may reflect increased crosslink density and network stiffness, potentially limiting cell spreading, nutrient diffusion, or effective encapsulation efficiency. Importantly, however, none of the formulations exhibited overt cytotoxicity, as all supported sustained cell viability and proliferation up to day 7. These findings are consistent with previous reports in the literature identifying an intermediate MA feed ratio (~3.5) as optimal for balancing crosslink density, mechanical stability, and cytocompatibility in injectable gelatin-based systems [11]. Similar to their observations, excessively high anhydride contents did not improve biological performance despite potentially increasing stiffness, underscoring that maximal crosslinking is not synonymous with optimal biofunctionality. Collectively, the present data confirm that moderate anhydride contents in the oSMoMA series provide the most favorable balance between network integrity and cellular compatibility for bioink applications.

Considering all characterization data for the gelatin hydrogels in this study, oSMoMA-3.5 consistently demonstrated the most promising properties for use as a bioink. Based on these findings, oSMoMA-3.5 was selected for subsequent bioprinting experiments. The frequency-dependent rheological behavior of the fully crosslinked and equilibrium-swollen bioink formulations is shown in Figure S5 (Supplementary Material).

### 2.4. Progressive Cavity Pump-Controlled Extrusion and Cytocompatibility

Murine 3T3 fibroblasts were encapsulated within gelatin/oSMoMA-3.5 formulations and processed using an extrusion-based printing setup operating on the endless piston principle. 3D printing of the cell-laden hydrogels was performed under sterile conditions using an extrusion-based setup integrated into an FDM printer chassis. An illustration of the bioprinting setup is shown in the Supplementary Material (Figure S5). However, in contrast to thermoplastic FDM processes, no drying or solidification of the printed material by cooling occurs. Instead, structural stabilization is achieved by chemical gelation via amine–anhydride crosslinking after deposition, with constructs incubated at 37 °C for 20 min under humidified conditions (5% CO_2_) to complete network formation before fresh cell culture medium was added. Hydrogels were printed directly onto sterile 13 mm PETG coverslips placed on a glass substrate to allow transfer into 12-well plates for subsequent cell culture. The printed constructs consisted of rectilinear grid structures (12 × 12 mm) with an infill density of 15% and four stacked layers, fabricated at a printing speed of 14 mm s^−1^. This geometry was selected to provide a defined and reproducible internal architecture while enabling nutrient diffusion and cell proliferation within the scaffold. The resulting constructs were used as standardized model systems for evaluating printability, cytocompatibility, and post-printing stability; no specific end-use application was intended within the scope of this work.

**Figure 7:**
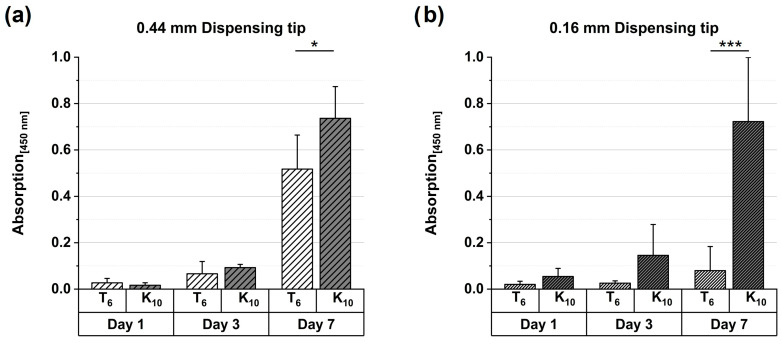
Metabolic activity of cells cultured in printed hydrogels, assessed as absorbance at 450 nm on days 1, 3, and 7. Hydrogels were printed using dispensing tips with diameters of (**a**) 0.44 mm and (**b**) 0.16 mm. Two formulations (TEOA 6% and K_2_HPO_4_ 10%) were evaluated. Measurements were performed in quadruplicate (*n* = 4). Bars represent mean values, and error bars indicate the standard deviation. Statistical differences between the base formulations on day 7 (*p* values shown in the graph) were determined by one-way ANOVA performed separately for each dispensing tip diameter, followed by Tukey’s post hoc test. (* represents *p* = 0.02 and *** represents *p* < 0.001).

To assess the influence of base type and dispensing tip diameter on cell viability, WST-8 assays were performed on constructs printed from two bioink formulations (6% TEOA and 10% K_2_HPO_4_) using dispensing tips of 0.44 mm and 0.16 mm diameter. For both formulations and both tip sizes, absorbance values increased from day 1 to day 7, indicating sustained viability and proliferation of embedded cells (Figure 7). These results confirm the general suitability of both bioinks for cell encapsulation and extrusion-based processing.

From day 3 onward, higher metabolic activity was observed for the K_2_HPO_4_-based formulation compared to the TEOA-based system, independent of tip diameter. Notably, the TEOA formulation exhibited a pronounced dependence on dispensing tip size at day 7: while constructs printed with the 0.44 mm tip showed a clear increase in absorbance, only a modest rise was detected for the 0.16 mm tip. This suggests that extrusion through the smaller tip imposed higher shear stress on embedded cells, adversely affecting their recovery and proliferation. Importantly, gelation kinetics differed between the two systems: the K_2_HPO_4_-based formulation exhibited slower gelation than the TEOA-based system. At comparable time points during dispensing, the faster-gelling TEOA formulation may therefore have exhibited higher viscosity within the dispensing tip, increasing shear exposure particularly in the smaller nozzle. In contrast, the slower gelation of the K_2_HPO_4_ formulation likely maintained a lower effective viscosity during extrusion, contributing to its reduced sensitivity to nozzle diameter and improved robustness toward shear stress. Overall, both systems supported cell proliferation up to day 7, but the data indicate that the TEOA-based bioink benefits from larger dispensing tip diameters, whereas the K_2_HPO_4_ formulation demonstrates greater tolerance toward extrusion-induced mechanical stress.

**Figure 8:**
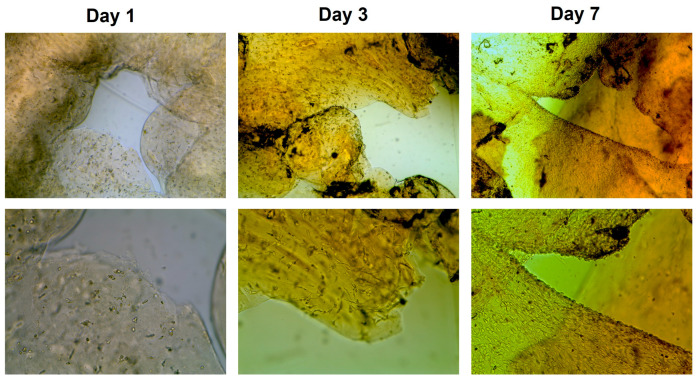
Light micrographs of murine 3T3 fibroblasts encapsulated in K_2_HPO_4_ (10%) bioink constructs printed with a 0.44 mm dispensing tip over a 7-day cultivation period. Representative regions of the printed grid structure are shown for days 1, 3, and 7. Images were acquired using 10× and 20× objectives (top and bottom rows, respectively).

In addition to quantitative metabolic measurements, transmitted light microscopy was performed to assess cell distribution and morphology within the K_2_HPO_4_ (10%) bioink (Figure 8). Constructs printed with a 0.44 mm dispensing tip were analyzed over a 7-day cultivation period. Consistent with the assay results, an increasingly intense orange coloration was observed over time, reflecting enhanced reduction in the WST-8 reagent and thus a rising number of metabolically active cells. Microscopic evaluation revealed a progressive increase in cell density within the hydrogel matrix over the cultivation period. Whereas distinct cell-free regions and only sparse cell distribution were apparent on day 1, the constructs appeared densely populated by day 7, with previously unoccupied regions largely filled. The initially observed gaps reflect the grid-like architecture of the printed constructs rather than an absence of cell attachment. Higher magnification images (40× objective) shown in Figure A2 confirm the presence of well-spread fibroblasts exhibiting the characteristic elongated and flattened morphology with irregular contours typical of adherent 3T3 cells. The cells formed contacts with neighboring cells, resulting in locally altered shapes and the emergence of interconnected cellular structures. The predominance of spread and adherent cells within the hydrogel indicates active cell–matrix and cell–cell interactions, supporting the conclusion of sustained viability and proliferation derived from the WST-8 analysis.

In summary, the cytocompatibility of the developed gelatin/oSMoMA hydrogels arises from the combined effects of formulation and crosslinking chemistry. In particular, the reduction in DMSO content to minimal levels limits solvent-induced cytotoxicity during and after gelation. In parallel, the use of an intermediate MA feed ratio (oSMoMA-3.5) provides a balanced crosslink density that ensures sufficient mechanical stability without excessive network stiffness. The amine–anhydride crosslinking proceeds under mild conditions, avoiding harsh triggers or reactive intermediates that could compromise cell viability. In addition, the gelatin matrix offers intrinsic cell-interactive motifs that support adhesion and proliferation of embedded fibroblasts. In contrast, higher MA contents are expected to increase crosslink density and stiffness, which may restrict cell spreading and impair nutrient diffusion within the network, thereby negatively affecting cellular activity. Together, these factors explain the observed high metabolic activity and sustained proliferation in the optimized formulations.

From a translational perspective, the degradation of gelatin/oSMoMA hydrogels is governed by the interplay of enzymatic and hydrolytic processes. Enzymatic degradation of the gelatin matrix is expected to dominate under physiological conditions, enabling host–cell infiltration and progressive tissue remodeling, while minor hydrolytic cleavage may further contribute to mass loss over time. The amine–anhydride-derived amide linkages are hydrolytically stable; however, overall network integrity decreases as the surrounding gelatin matrix is degraded. In addition, the gradual release of low-molecular-weight or partially hydrolyzed oligomer fractions may contribute to network erosion and influence long-term stability. As a result, a time-dependent reduction in mechanical properties, particularly storage modulus, can be anticipated. Similar degradation behavior has been reported for related two-component systems, which exhibit slow hydrolytic degradation but rapid enzymatic breakdown and complete in vivo resorption within weeks. Together, these considerations position the present system as a platform combining initial structural stability with biologically mediated degradation, although dedicated long-term studies are required to quantitatively assess degradation kinetics and their impact on tissue integration. Accordingly, in vivo performance of the gelatin/oSMoMA bioink will depend on the balance between scaffold degradation and tissue regeneration. Enzymatic degradation of the gelatin matrix is expected to enable host–cell infiltration and subsequent extracellular matrix deposition, gradually replacing the scaffold. Achieving a suitable degradation–regeneration balance is critical, as overly rapid degradation may compromise mechanical integrity, whereas excessive stability may hinder tissue remodeling. Future studies should therefore focus on correlating degradation kinetics, mechanical evolution, and biological response in relevant in vivo models.

Current bioinks for extrusion-based bioprinting are predominantly based on natural polymers such as alginate, gelatin, collagen, fibrin, or hyaluronic acid, as well as synthetic matrices including polyethylene glycol (PEG) derivatives [29]. Each class addresses specific requirements of printability, structural stability, and cytocompatibility, yet none satisfies all three criteria. Ionic systems such as alginate enable rapid gelation and excellent shape fidelity but often lack intrinsic cell-adhesion motifs and exhibit limited control over long-term mechanical properties. Protein-based matrices such as gelatin and collagen provide a biologically favorable microenvironment but typically suffer from insufficient mechanical stability and require additional crosslinking strategies. Photocrosslinkable systems such as methacrylated gelatin overcome this limitation by enabling tunable covalent network formation; however, they introduce additional complexity through the use of photoinitiators and light exposure, which may influence cell viability and complicate process control.

The bioink platform presented here addresses these limitations through a hybrid design that combines a natural polymer with a synthetic, anhydride-functional oligomer crosslinker. For a comparison of the bioink concept presented here to established bioinks please refer to Table S1 (Supplementary Material) [30,31,32]. In contrast to ionic gelation, the amine–anhydride reaction yields a covalently crosslinked network with improved structural stability and control of crosslinking kinetics, while avoiding the need for external triggers, such as photochemical activation required by many modern bioinks. At the same time, the oligomer composition enables systematic tuning of reactivity and network density, thereby providing a route to adjust mechanical properties without sacrificing cytocompatibility. The demonstrated compatibility of the oligomer crosslinker with multiple amine-containing biopolymers further highlights the versatility of the approach. Within this framework, gelatin-based formulations emerged as the most suitable system, combining favorable rheology, printability, and cell compatibility.

## 3. Conclusions

In this work, a two-component hydrogel platform based on amine–anhydride conjugation was successfully developed and validated as a cytocompatible bioink for extrusion-based 3D bioprinting. A new generation of anhydride-containing oligomers (oSMoMA-x) was designed by combining maleic anhydride as the reactive crosslinking moiety with stearylacrylate and the hydrophilic comonomer acryloylmorpholine. This molecular design enabled systematic tuning of oligomer composition and hydrophilicity, resulting in reduced cytotoxicity while maintaining sufficient reactivity toward amino-functional biopolymers.

The synthesized oSMoMA-x oligomers exhibited controlled molecular weights, high anhydride integrity, and predictable composition trends across the series. Their versatility was demonstrated by the successful formation of hydrogels with different natural polymers, including chitosan, gelatin, and HCP. Comparative evaluation revealed that gelatin-based hydrogels provided the most favorable balance between mechanical stability, processability, and cytocompatibility, leading to their selection for further bioink development.

Optimization of the gelatin/oSMoMA-3.5 system highlighted the critical influence of formulation parameters, particularly the concentration of DMSO and base, on cell viability and gelation behavior. By reducing the DMSO content and adjusting component ratios, two bioink formulations with distinct gelation kinetics and stiffness were obtained. Both bioinks supported high cell viability, exceeding that of a standard collagen hydrogel, and maintained cytocompatibility for up to 7 days.

Finally, the optimized bioinks were successfully processed using extrusion-based 3D bioprinting under physiological temperature conditions. Printed constructs supported cell survival and proliferation over at least seven days, confirming the suitability of the developed system for biofabrication. Overall, this study demonstrates that amine–anhydride-crosslinked gelatin/oSMoMA hydrogels represent a promising and adaptable bioink platform for 3D bioprinting in tissue engineering. By enabling tunable covalent crosslinking under mild conditions while maintaining favorable rheological properties and cytocompatibility, the system offers a means to balance the often competing requirements of controlled gelation, structural stability, and cell compatibility in extrusion-based bioprinting.

## 4. Materials and Methods

### Materials

4-Acryloylmorpholine (acryloylmorpholine, AMo), 2,2′-azobis(2-methylpropionitrile) (azobisisobutyronitrile, AIBN), chitosan low molecular weight (batch #STBH2613), dimethyl sulfoxide (anhydrous, ≥99.9%, DMSO), dimethyl sulfoxide-d6 (99.9%, DMSO-d6), gelatin from porcine skin type A bloom 300 (batch #SLCK3991), β-glycerophosphate disodium salt, octadecyl acrylate (stearyl acrylate, SA), picrylsulfonic acid solution 5% (*w*/*v*) in H_2_O (2,4,6-trinitrobenzenesulfonic acid solution, TNBS), 1,2-propanediol (propylene glycol, PG), hydrochloric acid (≥37%, HCl), and triethanolamine (≥99.0%, TEOA) were purchased from Sigma-Aldrich^®^ (Seelze, Germany). Aniline (99.5%), 3-hydroxy-1-methylpiperidine (NMPO), and maleic anhydride (99%, MA) were obtained from Thermo Fisher Scientific/Life Technologies GmbH (Darmstadt, Germany). Dulbecco’s Modified Eagle Medium with low glucose (DMEM), fetal bovine serum (FBS), penicillin/streptomycin (PenStrep), and 0.05% trypsin-EDTA (1×) were purchased from Gibco (Thermo Fisher Scientific/Life Technologies GmbH, Darmstadt, Germany). Dichloromethane, diethyl ether, ethanol (96% *v*/*v*), sodium hydroxide (NaOH), and 0.1 M sodium hydroxide solution were obtained from Fisher Scientific™ (Schwerte, Germany). Trypan blue solution (0.4%) for cell counting, and WST-8 cell viability reagent (Rotitest^®^ Vital) were supplied by Carl Roth GmbH + Co. KG (Karlsruhe, Germany). Benzoic acid (analytical grade), potassium hydrogen phthalate, sodium bicarbonate (NaHCO_3_), and tetrahydrofuran (THF; refluxed over sodium and potassium and freshly distilled prior to use) were obtained from Merck KGaA (Darmstadt, Germany). Dulbecco’s phosphate-buffered saline (DPBS) was obtained from PAN-Biotech GmbH (Aidenbach, Germany), and hydrolyzed collagen peptides (HCP) (Imagel^®^ batch #660057, now Peptiplus^®^ XB-UHV) was supplied by Gelita AG (Eberbach, Germany). Dipotassium hydrogen phosphate trihydrate and 0.1 M hydrochloric acid were obtained from VWR BDH Prolabo (Darmstadt, Germany). Demineralized ultrapure water used for all experiments in this study was produced using a MicroPure water purification system (Barnstead™ MicroPure™, Thermo Fisher Scientific/Life Technologies GmbH, Darmstadt, Germany).

**Oligomeric Crosslinker Synthesis and Characterization**

In accordance with previously reported protocols [11,14], the oligomeric crosslinkers oligo(SA-*ran*-AMo-*ran*-MA) (oSMoMA-x) were synthesized with different anhydride contents. Copolymerization was performed by free radical polymerization of comonomer mixtures of stearyl acrylate (SA), acryloylmorpholine (AMo) and maleic anhydride (MA) in THF at 60 °C under nitrogen atmosphere, using AIBN as the initiator. The molar ratio of SA to the comonomers (MA + AMo) was fixed at 1:20 for all synthesis variations. The molar ratio of MA to SA, indicated by *x* in the nomenclature, was preset between 2 and 10, resulting in a series of oSMoMA-x oligomers with different molar compositions (Table A1). After synthesis, the oligomers were precipitated using diethyl ether and subsequently dried under vacuum. Chemical composition, molecular weight distribution, and the proportion of intact anhydride functionalities were determined by titration following a previously reported method [14]. For ^1^H NMR analysis, the oligomer was dissolved in deuterated DMSO at a concentration of 2.5 mg/mL GPC samples were prepared in THF at a concentration of 10 mg/mL and analyzed as previously described [14]. Prior to use in crosslinking, oligomer batches were incubated in an oven at 130 °C for 24 h to enhance anhydride reactivity, as recommended in the literature [33].

**Fabrication of Crosslinked Hydrogels with Chitosan, HCP (Imagel^®^) and Gelatin**

All three biopolymers, gelatin, HCP, and chitosan, were prepared using modifications of previously published protocols [6,11,12], with the objective of improving either cytocompatibility, handling properties, or formulation reproducibility.

Gelatin (type A, Bloom 300) was dissolved in demineralized water (8.1% *w*/*v*) and subsequently diluted with DMEM at a 2:1 ratio to obtain a final concentration of 5.4% (*w*/*v*). All gelatin solutions were incubated at 60 °C for 1 h to reduce viscosity prior to sterile filtration through a 0.2 µm polyethersulfone membrane (Filtropur, Sarstedt, Germany). Before use or further dilution, the solutions were equilibrated at 37 °C for 24 h in a water bath (Corio CD-B27, Julabo Labortechnik, Seelbach, Germany). For standard comparative experiments with the biopolymer components, Triethanolamine (TEOA, 6% *w*/*v*) was used as the base, whereas dipotassium hydrogen phosphate (K_2_HPO_4_, 10% *w*/*v*) was also applied for experiments in which gelatin hydrogel was intended for printing. Both bases were prepared in demineralized water. The oligomer oSMoMA-x was dissolved at 30% (*w*/*v*) in DMSO. The final component ratio (gelatin solution:base solution:oligomer solution) was 55:30:5 (*v*/*v*).

HCP (Imagel^®^; 37.5% *w*/*v*) and the base NMPO (1500 mM) were each dissolved in demineralized water. A premix was formed by combining the two solutions at a 4:1 ratio (HCP:base). The oligomer oSMoMA-x was separately dissolved in DMSO at 7% (*w*/*v*) and subsequently mixed with the premix at a 1:1 ratio to yield the final hydrogel (45:45 (*v*/*v*)).

Chitosan (3.3% *w*/*v*) was dissolved in 10 mL of 0.05 M HCl, stirred overnight, and subsequently autoclaved for 15 min at 121 °C and 2 bar (DX-45, Systec GmbH, Linden, Germany). After autoclaving, 8 mL of DMEM were added under ice cooling with stirring (MR Hei-Tec, Heidolph Instruments, Schwabach, Germany). To adjust the pH, 2 mL of β-glycerophosphate in DMEM (100 mg (*w*/*v*)) were added. The pH of the solution was monitored after each step (FiveEasy, Mettler Toledo, Columbus, OH, USA). The oligomer oSMoMA-x was dissolved at 20% (*w*/*v*) in a DMSO/propylene glycol mixture (7:3) and incorporated into the hydrogel. The final hydrogel was formulated at a volumetric ratio of 80:8 (chitosan solution:oligomer solution).

**Figure A1:**
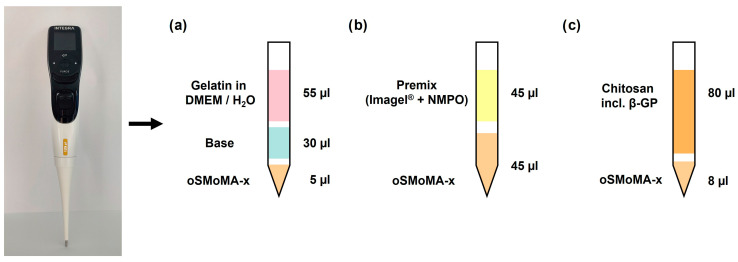
Photograph of the electronic programmable pipette used for hydrogel preparation. Schematic illustration of the tip filling for the fabrication of (**a**) gelatin-based, (**b**) HCP-based, and (**c**) chitosan-based two component hydrogels.

**Figure A2:**
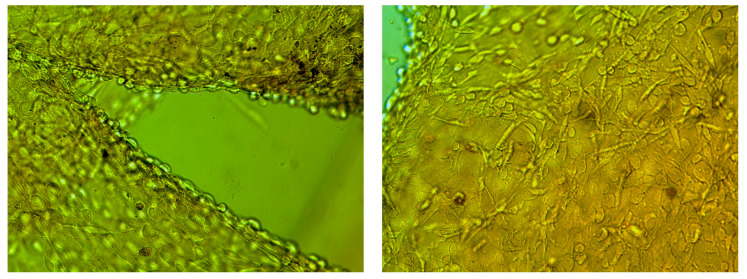
Light micrographs of murine 3T3 fibroblasts encapsulated in K_2_HPO_4_ (10%) bioink constructs printed with a 0.16 mm dispensing tip over a 7-day cultivation period. Representative regions of the printed grid structure are shown for day 7. Images were acquired using 40× objective.

For hydrogel formulations prepared at a total volume of 90 µL, components were combined at predetermined ratios using an electronic pipette to ensure immediate and uniform homogenization (Figure A1).

**Mechanical Characterization of the Hydrogels**

Based on previously reported protocols, the mechanical properties of the hydrogels were evaluated using oscillatory rheology [6]. Gelation kinetics of the crosslinking reaction were determined using a time-dependent oscillatory test on a Kinexus Pro rheometer (Netzsch, Selb, Germany). A 1°/20 mm cone was used as the upper geometry, and a 65 mm plate as the lower geometry. To prevent solvent evaporation during measurements, a solvent trap filled with demineralized water was employed. Hydrogel-forming components were deposited in predefined volumes and ratios onto the preheated (37 °C) lower plate using an electronic pipette. Measurements were initiated immediately at a frequency of 1 Hz and a strain amplitude of 0.05% for 1000 s. The storage modulus (G′), loss modulus (G″), and phase angle (δ) were recorded throughout the measurement. All experiments were performed in triplicate. For data analysis, both the full time-dependent curves and the storage modulus at 1000 s were considered.

To further evaluate the mechanical strength of the formed hydrogel plates, a frequency sweep test was performed. A plate-plate geometry was used, consisting of an 8 mm upper plate and a 65 mm lower plate. Hydrogels were rehydrated in PBS prior to testing and placed on the preheated (37 °C) lower plate. A normal force of 0.1 N was applied to avoid pre-damage of the samples. Frequency sweeps were conducted at a strain amplitude of 0.05% over a frequency range of 0.1–10 Hz. Storage modulus (G′), loss modulus (G″), and phase angle (δ) were recorded, and the storage modulus at 1 Hz was used to compare hydrogel stiffness. All measurements were performed in triplicate.

**Quantification of Leachables and Hydrogel Water Content**

Hydrogels on a larger scale (450 µL) were prepared in snap-cap vials, maintaining the defined ratios for each biopolymer. Following a previously reported protocol [6], the hydrogel discs were subjected to a series of drying and washing steps over 9 days. Lyophilization (Alpha 1–4 LDplus, Martin Christ Gefriertrocknungsanlagen GmbH, Göttingen, Germany) of the hydrated gels was performed to determine the fraction of leachable components (%). Water content was calculated from the wet weight of the 8 mm discs and the dry weight of the lyophilized large plates. All measurements were performed in triplicate.

**Crosslinking Degree and Cell Viability of Gelatin Hydrogels**

To complement the previously performed mechanical and compositional analyses, the gelatin hydrogels were additionally assessed for their crosslinking degree and cytocompatibility.

Crosslinking degree: The degree of crosslinking of the gelatin hydrogels was determined using a TNBS (2,4,6-trinitrobenzenesulfonic acid) assay, as described in previous protocols [21]. Lyophilized hydrogel samples (3 mg) were incubated with 1 mL 4% sodium bicarbonate, 4 mL demineralized water, and 1 mL 0.5% TNBS solution in the dark at 40 °C for 4 h. The reaction was terminated and the colored product stabilized by addition of 18 mL 6 N HCl, followed by incubation at 60 °C for 90 min. Blanks were treated identically, except that HCl was added prior to TNBS. Absorbance was measured at 346 nm using a 96-well plate reader (Spark, Tecan Group Ltd., Männedorf, Switzerland), and the crosslinking degree was calculated based on a standard curve prepared from non-crosslinked gelatin. The degree of crosslinking was calculated using the following formula:

$$Crosslinking\;degree\;\left[\%\right]=\;\left[1-\frac{\frac{Absorption}{Mass}\;\left(crosslinked\;sample\right)}{\frac{Absorption}{Mass}\;\left(reference\;gelatin\right)}\right]\times 100\%$$

Cell viability: The cytocompatibility of the gelatin hydrogels was assessed using the WST-8 assay on days 1, 3, and 7. Murine embryonic 3T3 fibroblasts were cultured in T75 polystyrene flasks (Sarstedt, Nümbrecht, Germany) in DMEM supplemented with 10% fetal bovine serum (FBS) and 1% penicillin/streptomycin. Prior to encapsulation, cells were detached and resuspended in fresh medium to a concentration yielding 180,000 cells per 90 µL hydrogel (2 × 10^6^ cells/mL). For hydrogel preparation, the cell suspension was mixed 1:2 with sterile 8.1% gelatin solution to achieve a final gelatin concentration of 5.4%. The hydrogel components- including the base and oligomer- were combined at a 55 + 30 + 5 µL ratio using an electronic pipette and cast into polypropylene molds within 24-well plates (Sarstedt, Nümbrecht, Germany). Hydrogels were allowed to gel at 37 °C in a humidified 5% CO_2_ incubator (CellXpert C170, Eppendorf, Hamburg, Germany) for 20 min. Subsequently, 1.2 mL of culture medium was added to each hydrogel, with 1 mL replaced after 1 h. Medium was refreshed every 48 h. Blank controls contained medium only and were treated identically. Prior to WST-8 measurement, 1 mL of medium was removed and 800 µL of fresh medium added, followed by incubation with 100 µL of 10% (*v*/*v*) WST-8 reagent for 90 min at 37 °C and 5% CO_2_. 100 µL of the supernatant were transferred to a 96-well plate, and absorbance was measured at 450 nm using a multimode plate reader (Spark, Tecan, Männedorf, Switzerland). Experiments were performed in quadruplicate for samples and in duplicate for blanks, and blank absorbance was subtracted from the sample values.

**3D Bioprinting Setup**

For hydrogel printing experiments, a Creality CR-20 Pro printer (Shenzhen Creality 3D Technology, Shenzhen, China) was used. To allow precise volumetric extrusion of 9.4 µL per rotation and enable hydrogel printing instead of standard filament, the original printhead was replaced with one or two Puredyne^®^ printheads (ViscoTec Pumpen- u. Dosiertechnik GmbH, Töging am Inn, Germany). The printer was equipped with a Bigtreetech SKR 2 mainboard (Shenzhen Bigtree Technology, Shenzhen, China) for proper control of the new printheads. For temperature-controlled printing, a Puredyne^®^ temperature control unit (b5) was used, maintaining the printhead at 37 °C via a laboratory power supply (PS2107, Sky TopPower, Shenzhen, China). The setup for extrusion-based bioprinting is depicted in the Supplementary Material (Figure S5).

**3D Bioprinting of Cell-Laden Gelatin Hydrogels**

The 3D bioprinter and all cell-contacting components were sterilized with 70% (*v*/*v*) isopropanol or by autoclaving (DX-45, Systec GmbH, Linden, Germany) and assembled under a sterile laminar flow hood (ScanLaf Mars, LaboGene, Allerød, Denmark). Murine embryonic 3T3 fibroblasts were prepared as described for the cell viability assays and resuspended in 400 µL DMEM to a final concentration of 2 × 10^6^ cells/mL. The cell suspension was mixed with 800 µL sterile 8.1% gelatin solution to achieve a final gelatin concentration of 5.4%. From this mixture, 1100 µL were combined with 600 µL sterile base solution and 100 µL sterile oligomer solution (oSMoMA-3.5; 30% in DMSO) in the printer cartridge and transferred to the preheated (37 °C) printhead of the Puredyne^®^ system (ViscoTec Pumpen- u. Dosiertechnik GmbH, Töging am Inn, Germany). Hydrogels were printed under the laminar flow hood onto sterile 13 mm PETG coverslips (Carl Roth GmbH + Co. KG, Karlsruhe, Germany) placed on sterile glass plates to facilitate handling and transfer into 12-well plates (Cole-Parmer, Vernon Hills, IL, USA). Two micro-precision dispensing needles with different inner diameters (0.44 mm, 25 G and 0.16 mm, 34 G; Super Micro Dosiernadel, Vieweg GmbH, Kranzberg, Germany) were used for printing. Each hydrogel formulation was printed in eight replicates per needle size. Cell-free controls were printed in duplicate using DMEM only. Following printing, cell viability was assessed using the WST-8 assay and hydrogels were examined by brightfield microscopy.

**Cell Viability of 3D-Printed Gelatin Hydrogels**

After printing, the cell-laden hydrogels on the coverslips were transferred into 12-well plates (Cole-Parmer, Vernon Hills, IL, USA) and allowed to gel for 20 min at 37 °C and 5% CO_2_ (CellXpert C170, Eppendorf, Hamburg, Germany). Subsequently, 2 mL of culture medium were added to each hydrogel, with 1 mL replaced after 1 h and the medium refreshed every 48 h thereafter. Prior to WST-8 assay, 1 mL of medium was removed and replaced with 1 mL fresh medium, followed by addition of 200 µL WST-8 reagent (10% *v*/*v*). The assay was then conducted as described above for the standard cell viability measurements. Hydrogels were also examined under brightfield microscopy (Leica DM IL LED, Leica Microsystems GmbH, Wetzlar, Germany) at 1, 3, and 7 days using 10× and 20× objectives to monitor cell morphology.

## Figures and Tables

**Table 1:** Hydrogel compositions identified for each investigated natural biopolymer showing the most favorable rheological and physicochemical properties. Refer also to the pipetting scheme illustrated in Figure A1.

Hydrogel Component	Gelatin	HCP (Imagel^®^)	Chitosan
Biopolymer solution (BPS)	5.4% in DMEM/water	37.5% in water	3.3% in 0.05 M HCl
Base	6% TEOA in water	1500 mM NMPO added to BPS	0.5% β-glycerophosphate added to BPS
Oligomer solution	oSMoMA-3.5 (30% in DMSO)	oSMoMA-7.5 (7% in DMSO)	oSMoMA-5 (40% in DMSO: propylene glycol (7:3))
Fractions [µL] per gel BPS (+Base) + Oligomer sol.	55 + 30 + 5	45 + 45	80 + 8

## Data Availability

Dataset available on request from the authors.

## Supplementary Materials

The following supporting information can be downloaded at: https://www.mdpi.com/article/10.3390/gels12050437/s1, Figure S1: Proton NMR spectrum of oSMoMA-5; Figure S2: Effect of TEOA concentration on hydrogel stiffness; Figure S3: Effect of K_2_HPO_4_ concentration on hydrogel stiffness; Figure S4: Evaluation of DMSO and oligomer cytotoxicity toward murine embryonic 3T3 fibro-blasts; Figure S5: Frequency-dependent rheological properties of the two bioink formulations after crosslinking into homogeneous, equilibrium-swollen hydrogels; Figure S6: Experimental setup for extrusion-based bioprinting; Table S1: Comparative Overview of Representative Bioink Systems and Key Performance Metrics.

## Abbreviations

The following abbreviations are used in this manuscript:

AIBN	Azobisisobutyronitrile
AMo	Acryloylmorpholine
β-GP	β-Glycerophosphate
BFT	Brown–Fujimori titration
CO_2_	Carbon dioxide
D	Dispersity (molecular weight)
DAAm	Diacetone acrylamide
DMF	Dimethylformamide
DMEM	Dulbecco’s Modified Eagle Medium
DMSO	Dimethyl sulfoxide
ECM	Extracellular matrix
FBS	Fetal bovine serum
G′	Storage modulus
G″	Loss modulus
GPC	Gel permeation chromatography
h	Hours
HCl	Hydrochloric acid
HCP	Hydrolyzed collagen peptides
H_2_O	Demineralized water
Hz	Hertz
K_2_HPO_4_	Dipotassium hydrogen phosphate
MA	Maleic anhydride
Mn	Number-average molecular weight
Mw	Weight-average molecular weight
N	Normality
NaOH	Sodium hydroxide
NIPAm	N-isopropylacrylamide
nm	Nanometers
NMPO	3-hydroxy-1-methylpiperidine
NMR	Nuclear Magnetic Resonance spectroscopy
N_2_	Nitrogen
oPDMA	Oligo(PEDAS-*co*-DAAm-*co*-MA)
oPNMA	Oligo(PEDAS-*co*-NIPAm-*co*-MA)
oSMoMA	Oligo(SA-*co*-AMo-*co*-MA)
Pa	Pascal
PBS	Phosphate-buffered saline)
PEDAS	Pentaerythritol diacrylate monostearate
PG	Propylene glycol
s	Seconds
SA	Stearyl acrylate
TEOA	Triethanolamine
THF	Tetrahydrofuran
TNBS	Trinitrobenzenesulfonic acid
WST	Water-soluble tetrazolium

## Appendix A

**Table A1:** Molar monomer feed ratios of stearyl acrylate (SA), maleic anhydride (MA), and acryloylmorpholine (AMo) used for the synthesis of oligo(SA-co-AMo-co-MA) (oSMoMA-x).

oSMoMA Type	Stearyl Acrylate	Acryloylmorpholine	Maleic Anhydride
oSMoMA-2	1	18	2
oSMoMA-2.5	1	17.5	2.5
oSMoMA-3	1	17	3
oSMoMA-3.5	1	16.5	3.5
oSMoMA-4	1	16	4
oSMoMA-5	1	15	5
oSMoMA-6	1	14	6
oSMoMA-7.5	1	12.5	7.5
oSMoMA-10	1	10	10
