# Supplementary material for: Hydrophilic Anhydride-Containing Oligomers for Two-Component Hydrogels: From Biopolymer Compatibility to Cytocompatible Gelatin Bioinks
Source: Gels. 2026 May 16;12(5):437. doi: 10.3390/gels12050437 (PMC13205935; doi:10.3390/gels12050437)
Supplement: Supplementary file 1 [file gels-12-00437-s001.zip › gels-4254437-supplementary.pdf]

Supplementary Material

Hydrophilic Anhydride-Containing Oligomers for Two-Component Hydrogels: From Biopolymer Compatibility to Cytocompatible Gelatin Bioinks

Julia C. Matros [1], Katharina E. Wiebe-Ben Zakour [2], Joana Witt [2] and Michael C. Hacker [1,*]

[1] Heinrich Heine University Düsseldorf, Faculty of Mathematics and Natural Sciences, Institute of Pharmaceutics and Biopharmaceutics, 40225 Düsseldorf, Germany
[2] Department of Ophthalmology, Medical Faculty and University Hospital Düsseldorf, Heinrich Heine University Düsseldorf, 40225 Düsseldorf, Germany
* Correspondence: michael.hacker@hhu.de (M.C.H.)

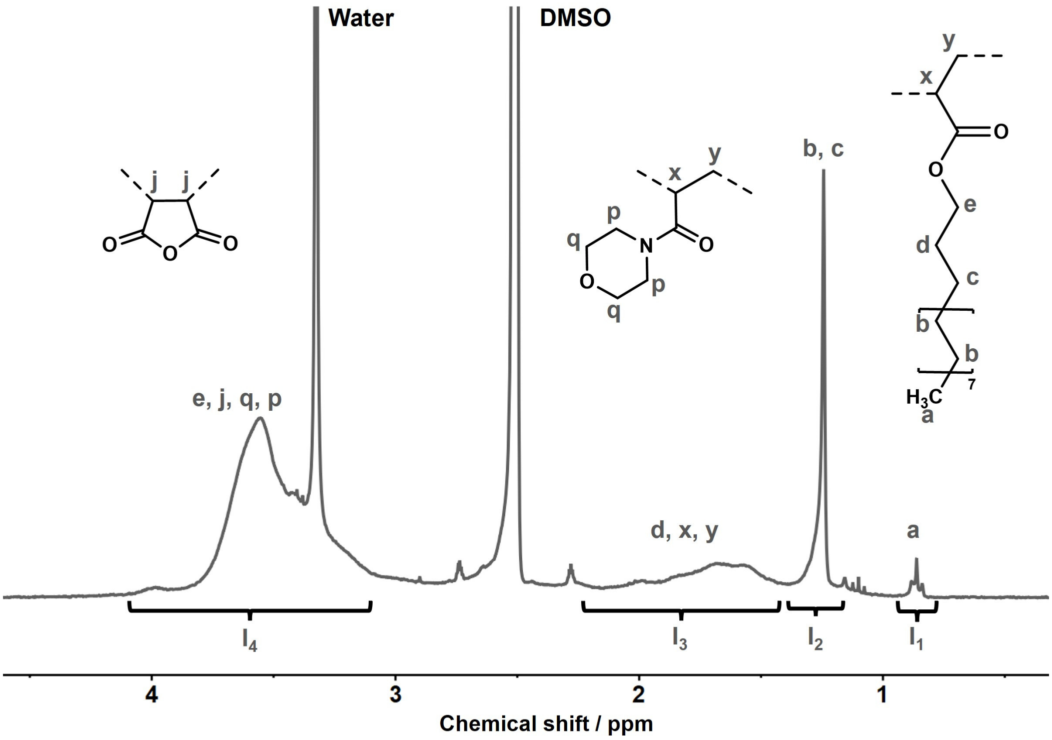

Figure S1: Proton NMR spectrum of oSMoMA-5 shown as a representative example of the oligomer series. Integrals I1–I4 correspond to characteristic proton signals used for determining the comonomer composition of the copolymer according to previous reports (Maqsood et al. https://doi.org/10.1039/D5TB02401C). The letters assigned to the integrals denote the protons of the respective structural units of the oSMoMA-x oligomer (maleic anhydride, acryloylmorpholine, and stearylacrylate), which were used to calculate the relative comonomer ratios.

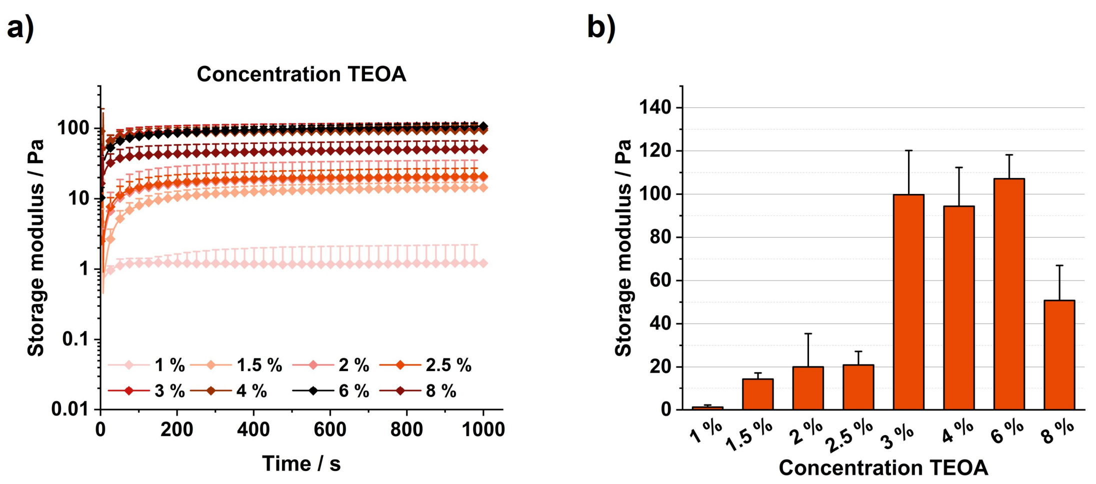

Figure S2: Effect of TEOA concentration on hydrogel stiffness. (a) Evolution of the storage modulus (G) during gelation over time. (b) Comparison of G' values measured at 1000 s. Increasing base concentration led to higher G' values and thus increased hydrogel stiffness, reaching a maximum at 6% TEOA. To further evaluate this trend, additional experiments were conducted at lower TEOA concentrations (1 – 2.5%). These results showed that effective gelation occurred only at concentrations $\geqslant$3%, whereas lower concentrations were insufficient to neutralize protons generated during the crosslinking reaction. At concentrations above the optimum (e.g., 8% TEOA), the storage modulus decreased again. Based on these observations, 6% TEOA was selected for subsequent experiments as it provided the highest and most stable G' while ensuring reliable hydrogel formation.

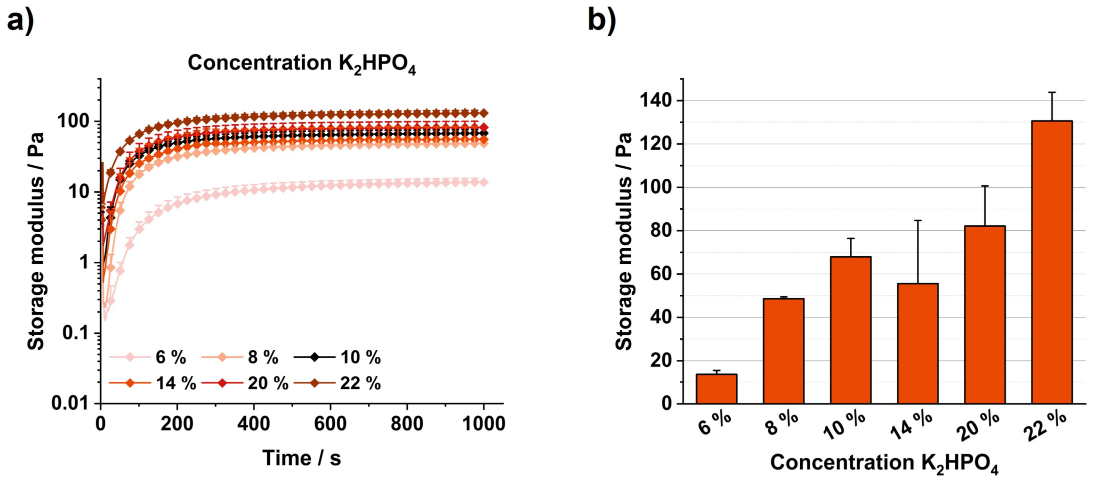

Figure S3: Effect of K_2HPO_4 concentration on hydrogel stiffness and optical properties (data not shown). (a) Evolution of the storage modulus (G) during gelation over time. (b) Comparison of G' values measured at 1000 s. Base concentrations ranging from 6% to 22% were evaluated. Increasing K_2HPO_4 concentration resulted in higher storage modulus (G) values and thus increased hydrogel stiffness. In contrast to TEOA, no saturation of G' was observed within the investigated concentration range. However, further increases were limited by precipitation effects during hydrogel preparation, which led to increasing turbidity of the gels. Slight turbidity appeared at 14% K_2HPO_4, while complete turbidity was observed at 22%. Similar turbidity upon addition of K_2HPO_4 has been reported previously, although in that case it could be resolved by short mixing periods [20]. Based on these observations, a concentration of 10% K_2HPO_4 was selected for subsequent experiments, as it provided high hydrogel stiffness while maintaining optical clarity and preventing precipitation.

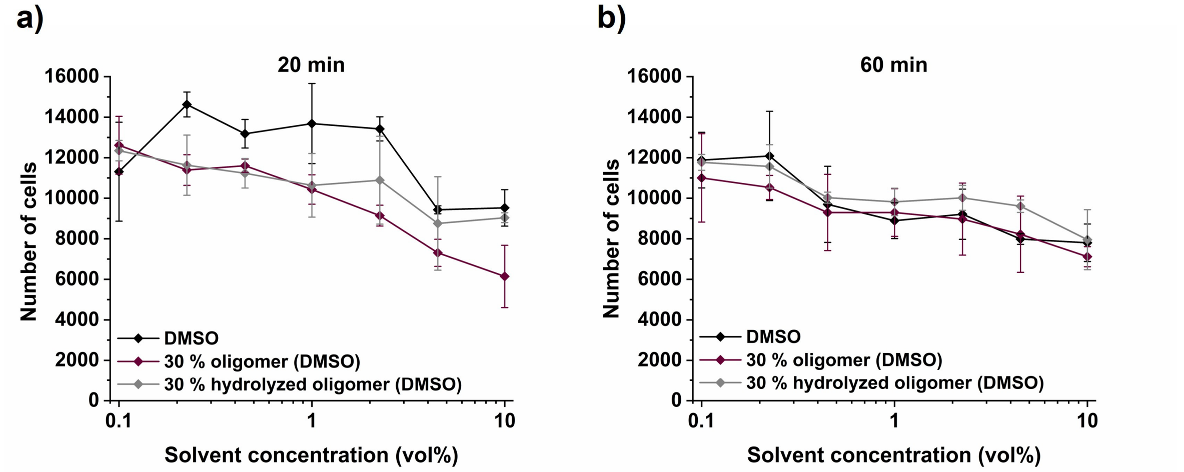

Figure S4: Evaluation of DMSO and oligomer cytotoxicity toward murine embryonic 3T3 fibroblasts. Cytotoxic effects of DMSO and oligomer-containing solutions were assessed because both components come into direct contact with cells during hydrogel preparation. Cell numbers were determined by a WST-8 assay using calibration. Different solvent concentrations and oligomer states (30% oligomer in DMSO and 30% hydrolyzed oligomer in DMSO) were tested, with concentrations expressed as volume percentages in culture medium (DMEM). Exposure times of (a) 20 and (b) 60 min were chosen to represent the approximate gelation period after hydrogel preparation (20 min) and the time point of the first medium exchange (60 min), when DMSO and soluble hydrogel components are largely removed. Tested concentrations covered the relevant range around the final DMSO content of the bioink formulation (5.56%). Cell viability remained above 75% up to a concentration of 4% in all conditions. For both exposure times, increasing solvent concentration led to a progressive decline in cell number above ~1% solvent or oligomer solution. The presence of oligomer—both non-hydrolyzed and hydrolyzed—caused a slight additional decrease in viability. At 20 min exposure, the hydrolyzed oligomer exhibited lower cytotoxicity than the non-hydrolyzed form, likely due to the absence of intact anhydride groups that can react with cellular amine groups via amine–anhydride conjugation.

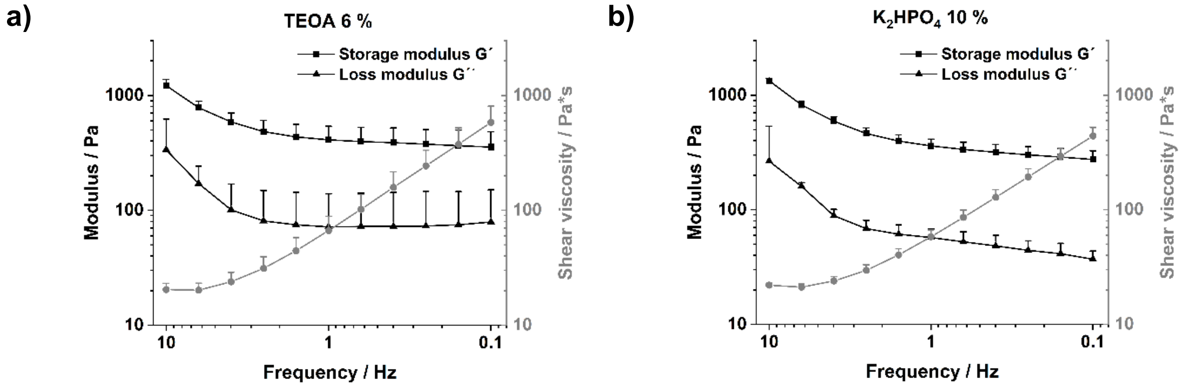

Figure S5: Frequency-dependent rheological properties of the two bioink formulations after crosslinking into homogeneous, equilibrium-swollen hydrogels: (a) gelatin/oSMoMA-3.5 with 6% TEOA and (b) gelatin/oSMoMA-3.5 with 10% potassium phosphate. The frequency sweep indicates a predominantly elastic network, as evidenced by storage modulus (G′) exceeding loss modulus (G″) across the entire frequency range, characteristic of a stable, crosslinked hydrogel. The weak frequency dependence of G′ suggests a well-developed network with quasi-permanent junctions, while the absence of a G′/G″ crossover confirms solid-like behavior. The decrease in both moduli at lower frequencies reflects viscoelastic relaxation processes and time-dependent network rearrangements. The increase in complex viscosity toward lower frequencies is consistent with long relaxation times and

high network connectivity. Although the frequency-dependent viscosity trend may qualitatively suggest shear-thinning behavior, this cannot be conclusively established without complementary steady-shear measurements.

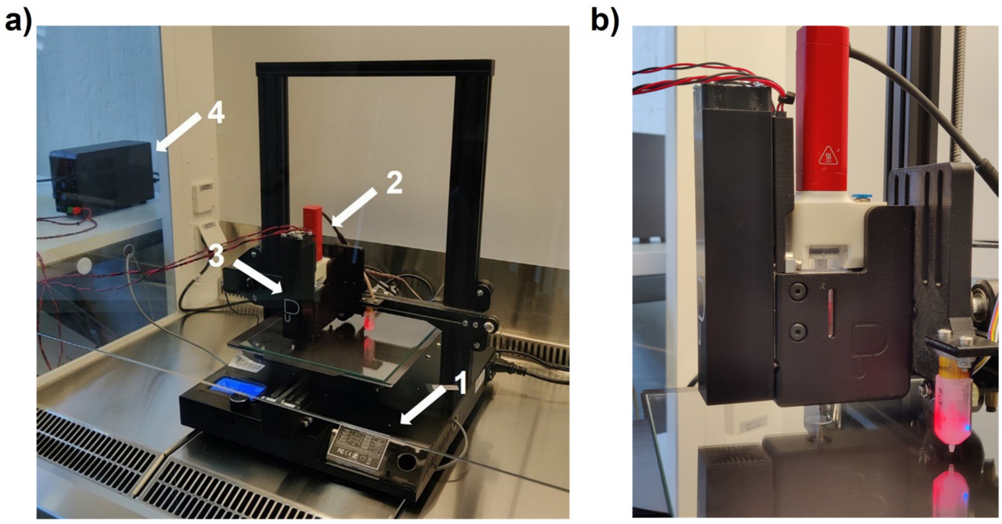

Figure S6: Experimental setup for extrusion-based bioprinting. (a) Printing system comprising (1) Creality CR-20 Pro printer, (2) Puredyne® printhead, (3) temperature control unit, and (4) laboratory power supply used to regulate the temperature unit. (b) Puredyne® printhead mounted within the temperature control unit.

Table S1: Comparative Overview of Representative Bioink Systems and Key Performance Metrics

Bioink system	*Crosslinking / stabilization*	*External trigger*	*Typical mechanical / rheological profile*	*Reported cell compatibility*	*Main strengths*	*Main limitations*
**Alginate-based bioinks**	Ionic crosslinking with Ca^{2+}	Ca^{2+} bath or Ca^{2+} release	Rapid gelation; stiffness tunable by alginate/Ca^{2+} concentration	Generally high viability; alginate is widely used due to biocompatibility and fast gelation	Excellent initial shape fidelity, simple processing	Limited intrinsic cell adhesion; limited biological remodeling; long-term mechanics may change
**Alginate/ gelatin blends**	Ionic alginate crosslinking + thermo-responsive gelatin	Temperature + Ca^{2+}	Higher viscosity and storage modulus than alginate alone; improved extrusion fidelity	Reported as suitable for cell-laden extrusion; Alg-Gel formulations improve printability and consistency	Combines printability with gelatin bioactivity	Gelatin melting/thermal sensitivity; ionic network can relax over time
**GelMA bioinks**	Photocrosslinking of methacryloyl groups	UV/visible light + photoinitiator	Tunable stiffness via GelMA concentration	GelMA/gelatin constructs reported ~90% cell viability in	Bioactive gelatin backbone; tunable covalent network	Requires photoinitiator and light exposure;

			and degree of substitution; recommended extrusion viscosity ranges reported around 100–1000 Pa·s	representative studies		viscosity/printability trade-off
**PEG-based bioinks**	Synthetic covalent crosslinking, e.g., PEG crosslinkers or photo-click systems	Often light or chemical crosslinking	Highly tunable mechanics and reproducibility	Cytocompatibility can be optimized while tailoring post-printing mechanics	Defined chemistry, reproducibility	Usually lacks intrinsic cell-adhesion/degradation motifs unless functionalized
**Gelatin/ oSMoMA bioink, this study**	Amine–anhydride conjugation between gelatin amines and oSMoMA anhydrides	No photochemical trigger; base-assisted two-component mixing	Tunable gelation kinetics and G′ through oligomer/base composition; extrusion-compatible	Sustained 3T3 fibroblast metabolic activity/proliferation up to 7 days	Covalent crosslinking under mild conditions; gelatin bioactivity; no photoinitiator/light exposure	Long-term degradation, full flow curves/yield stress, and in vivo remodeling remain future work

Established bioinks cover a broad formulation space, including natural, synthetic, and hybrid materials, but the requirements of printability, structural stability, and cytocompatibility often remain difficult to satisfy simultaneously. Alginate-based systems are attractive because of rapid ionic gelation and biocompatibility, but lack intrinsic cell-adhesive motifs; GelMA provides a bioactive and enzymatically degradable gelatin-derived matrix with tunable photocrosslinking, but requires photoinitiators and light exposure; PEG-based systems offer high chemical definition and tunable mechanics but usually require additional biofunctionalization. Therefore, the gelatin/oSMoMA system occupies an intermediate position by combining gelatin bioactivity with tunable covalent crosslinking through amine–anhydride chemistry without photochemical activation.
